# Suberanilohydroxamic acid prevents TGF-β1-induced COX-2 repression in human lung fibroblasts post-transcriptionally by TIA-1 downregulation

**DOI:** 10.1016/j.bbagrm.2018.03.007

**Published:** 2018-05

**Authors:** Alice Pasini, Oliver J. Brand, Gisli Jenkins, Alan J. Knox, Linhua Pang

**Affiliations:** a Division of Respiratory Medicine, University of Nottingham School of Medicine, City Hospital, Nottingham NG5 1PB, United Kingdom; b Department of Electrical, Electronic and Information Engineering “Guglielmo Marconi” (DEI), University of Bologna, Via Venezia 52, 47521 Cesena, FC, Italy

**Keywords:** Pulmonary fibrosis, Cyclooxygenase 2 (COX-2), Post-transcriptional regulation, epigenetics, Histone deacetylase inhibitor, Transforming growth factor β1 (TGF-β1)

## Abstract

Cyclooxygenase-2 (*COX*-*2*), with its main antifibrotic metabolite PGE_2_, is regarded as an antifibrotic gene. Repressed *COX*-*2* expression and deficient PGE_2_ have been shown to contribute to the activation of lung fibroblasts and excessive deposition of collagen in pulmonary fibrosis. We have previously demonstrated that *COX*-*2* expression in lung fibroblasts from patients with idiopathic pulmonary fibrosis (IPF) is epigenetically silenced and can be restored by epigenetic inhibitors. This study aimed to investigate whether COX-2 downregulation induced by the profibrotic cytokine transforming growth factor-β1 (TGF-β1) in normal lung fibroblasts could be prevented by epigenetic inhibitors. We found that COX-2 protein expression and PGE_2_ production were markedly reduced by TGF-β1 and this was prevented by the pan-histone deacetylase inhibitor suberanilohydroxamic acid (SAHA) and to a lesser extent by the DNA demethylating agent Decitabine (DAC), but not by the G9a histone methyltransferase (HMT) inhibitor BIX01294 or the EZH2 HMT inhibitor 3-deazaneplanocin A (DZNep). However, chromatin immunoprecipitation assay revealed that the effect of SAHA was unlikely mediated by histone modifications. Instead 3′-untranslated region (3′-UTR) luciferase reporter assay indicated the involvement of post-transcriptional mechanisms. This was supported by the downregulation by SAHA of the 3′-UTR mRNA binding protein TIA-1 (T-cell intracellular antigen-1), a negative regulator of COX-2 translation. Furthermore, TIA-1 knockdown by siRNA mimicked the effect of SAHA on COX-2 expression. These findings suggest SAHA can prevent TGF-β1-induced COX-2 repression in lung fibroblasts post-transcriptionally through a novel TIA-1-dependent mechanism and provide new insights into the mechanisms underlying its potential antifibrotic activity.

**Abbreviations:**

**Table:** 

SAHA	suberanilohydroxamic acid
TGF-β1	transforming growth factor-β1
COX-2	cyclooxygenase-2
TIA-1	T-cell intracellular antigen-1
PGE_2_	prostaglandin E_2_
IPF	idiopathic pulmonary fibrosis
DAC	Decitabine
HMT	histone methyltransferase
EZH2	enhancer of zeste homolog 2
DZNep	3-deazaneplanocin A
3′-UTR	3′-untranslated region
α-SMA	α-smooth muscle actin
ECM	extracellular matrix
COL1	collagen 1
DNMT	DNA methyltransferase
HAT	histone acetyltransferase
HDAC	histone deacetylase
H3K9me3	histone H3 lysine 9 trimethylation
ARE	AUUUA-rich element
HuR	human antigen R
ELAV1	ELAV-like RNA binding protein 1
TTP	Tristetraprolin
CUGBP2	CUG triplet repeat, RNA binding protein 2
F-NL	fibroblast from non-fibrotic lung
FCS	fetal calf serum

## Highlights

• The HDAC inhibitor SAHA upregulates the expression of the antifibrotic gene *COX-2* post-transcriptionally.
• The mechanism relies on the downregulation of TIA-1, a negative regulator of COX-2 translation.
• SAHA has a therapeutic potential by preventing COX-2 repression induced by TGF-β1 in human lung fibroblasts.

## 1 Introduction

Activated lung fibroblasts (myofibroblasts) are characterized by their ability to express α-smooth muscle actin (α-SMA) and secrete extracellular matrix (ECM) proteins, particularly collagen 1 (COL1). They are regarded as key effector cells in pulmonary fibrosis, particularly in idiopathic pulmonary fibrosis (IPF), a fatal lung disease with unknown aetiology and a lack of specific effective therapies. Experimental evidence points to the profibrotic cytokine transforming growth factor-β (TGF-β1) as an important driving factor of fibrosis initiation and progression. TGF-β1 has been shown to induce fibroblast activation, excessive production of ECM and inhibition of ECM degradation [1]. Blocking TGF-β1 effectively reduced lung fibrosis in animal models, and inhibition of TGF-β1 signalling slowed the progression of IPF in patients [2]. But pan-TGF-β blocking shows lack of efficacy and causes pleiotropic effects.

Prostaglandin E_2_ (PGE_2_) is a potent antifibrotic mediator. It inhibits fibroblast-to-myofibroblast differentiation and many pro-fibrotic features of lung myofibroblasts, including proliferation, migration, and collagen production. However, both lung fibroblasts isolated from IPF patients and TGF-β1-activated lung fibroblasts have been shown to be associated with reduced PGE_2_ production as a result of down-regulation of cyclooxygenase-2 (COX-2), a rate-limiting enzyme responsible for PGE_2_ production [[3], [4], [5]]. Reduced COX-2 expression has also been observed in bronchial epithelial cells of IPF patients, suggesting that COX-2 downregulation is not limited to lung fibroblasts [6]. Furthermore, *COX*-*2*-deficient mice, having limited PGE_2_ synthesis, are more susceptible to bleomycin-induced pulmonary fibrosis [7]; in contrast, *COX*-*2* overexpression in the lung leads to increased PGE_2_ synthesis and reduced fibroblast proliferation [8]. These observations suggest that the antifibrotic COX-2/PGE_2_ mechanism is lost in fibrotic lung due to COX-2 repression.

Epigenetic regulation of gene expression is a key mechanism in the activation or silencing of genes. DNA methylation at CpG islands in gene promoter regions catalysed by DNA methyltransferases (DNMTs) is usually associated with gene silencing. Acetylation and deacetylation of histone lysine residues by histone acetyltransferases (HATs) and histone deacetylases (HDACs) are associated with transcriptional activation and repression, respectively. Methylation of lysine residues at histone H3 and H4 tails can be associated with either transcriptional activation or repression depending on the specific site and the number of methyl groups added. Trimethylation of H3 lysine 9 and 27 (H3K9me3, H3K27me3) by histone methyltransferase (HMT) G9a and EZH2 (enhancer of zeste homolog 2), respectively, are enriched in transcriptionally repressed promoter regions, whereas H3K4me3 by the Trithorax complex is enriched in active promoter regions [9]. We have previously reported that in lung fibroblasts from IPF patients, the *COX*-*2* promoter region is associated with repressive histone modifications, i.e. H3 and H4 deacetylation and H3K9 and H3K27 methylation. Furthermore, epigenetic inhibitors LBH589 (panobinostat, a pan-HDAC inhibitor), BIX02189 (a G9a inhibitor) or 3-deazaneplanocin A (DZNep, an EZH2 inhibitor), can restore *COX*-*2* expression and PGE_2_ production by reversing the repressive histone modifications [3,5].

Post-transcriptional mechanisms also play a critical role in regulating COX-2 expression, conferred by the conserved AUUUA-rich elements (AREs) located in the 3′-untranslated region (3′-UTR) of *COX-2* transcripts. AREs function to target mRNA for rapid decay or stabilization and to promote or inhibit translation, depending on the specific ARE binding proteins or microRNAs [10]. Different ARE binding proteins have been found to regulate *COX-2* post-transcriptionally, especially in colon cancer [11]. Among them, HuR (human antigen R), also known as ELAV-like RNA binding protein 1 (*ELAV1*), displays high affinity for AREs and stabilizes ARE-containing mRNAs and promotes their translation upon binding [12]. HuR is overexpressed in colon adenomas and adenocarcinomas and its ability to target 3′-UTR-mediated COX-2 upregulation has been demonstrated in colon cancer cells [13]. Tristetraprolin (TTP) has been reported to promote rapid mRNA decay [14] and is involved in COX-2 downregulation in colon cancer cells, driving *COX*-*2* mRNA for rapid degradation [13,15]. CUG triplet repeat, RNA binding protein 2 (CUGBP2), similarly to HuR, increases *COX*-*2* mRNA stability, but also inhibits COX-2 protein translation [16]. T-cell intracellular antigen-1 (TIA-1) has been shown to bind to ARE in the 3′-UTR of *COX-2* transcripts and functions as a translational silencer of COX-2 [15,17].

Suberanilohydroxamic acid (SAHA) (trade name Vorinostat), is a non-selective HDAC class 1 and 2 inhibitor and has been approved for the treatment of peripheral and cutaneous T-cell lymphoma by the Food and Drug Administration (FDA) of the US. It is under evaluation for the treatment of non-small cell lung cancer in combination with DNA demethylating agents and chemotherapy [18]. SAHA has been shown to abrogate TGF-β1-induced lung fibroblast activation and collagen expression [36] and significantly reduce collagen deposition in a murine model of bleomycin-induced pulmonary fibrosis [19,20], suggesting promising antifibrotic potential, however the underlying molecular mechanisms are not clear yet. Although SAHA, as a HDAC inhibitor, can regulate gene expression through transcriptional activation, there is evidence that it can also regulate gene expression post-transcriptionally by increasing mRNA stabilization [21] and suppressing protein translation [22]. However, how SAHA may modulate COX-2 expression and the release of the antifibrotic mediator PGE_2_ in TGF-β1-induced lung fibroblast activation remains to be clarified.

Since epigenetic inhibitors have been shown to restore *COX*-*2* expression and PGE_2_ production in lung fibroblasts from IPF patients by reversing the repressive histone modifications [3,5], the aim of this study was to evaluate the effect of these drugs on COX-2 downregulation associated with TGF-β1-induced activation of normal human lung fibroblasts. The results presented here demonstrate that the pan-HDAC inhibitor SAHA is the most promising among the inhibitors tested in upregulating COX-2 protein expression and PGE_2_ production and that its effect is mainly mediated via a novel post-transcriptional mechanism by supressing the expression of the translational repressor ARE binding protein TIA-1. This mechanism may contribute to the antifibrotic effect of SAHA.

## 2 Materials and methods

### 2.1 Fibroblast cell culture

Fibroblast from non-fibrotic lung (F-NL) obtained from the University of Pittsburgh Medical Center (Pittsburgh, PA, USA) were isolated from normal lung tissues from organ donors under a protocol approved by the University of Pittsburgh Institutional Review Board and cultured as described previously [23]. The four donors included two males and two with undisclosed gender; the age of one donor was undisclosed and the average age of the other three donors was 46.7 years (range, 27–63). One donor was a nonsmoker and the smoking history of the other three was undisclosed. The cells were grown to passage six in DMEM with 10% foetal calf serum (FCS) (complete DMEM) as described before [5]. They were seeded up to 24 h before starting the treatments to ensure an exponential growth phase. They were then pre-treated with the G9a inhibitor BIX01294 (BIX, 100 nM), the EZH2 inhibitor 3-Deazaneplanocin A (DZNep, 10 nM), the HDAC inhibitor suberanilohydroxamic acid (SAHA, 5 μM, Cayman Chemical, Ann Arbor MI, USA), the hypomethylating agent Decitabine (DAC, 1 μM, Biovision, Milpitas, CA, USA), or vehicle, for 1 h prior to incubation with recombinant human TGF-β1 (2 ng/ml, Peprotech, London, UK) for 96 h (48 h in complete DMEM followed by 48 h in serum-free DMEM), in combination with recombinant human IL-1β (1 ng/ml, Peprotech) for up to the last 24 h. At the indicated time points, cells were collected for subsequent analyses.

### 2.2 Quantitative real-time RT-PCR (qRT-PCR)

qRT-PCR was applied to analyze mRNA expression of genes. After 4 h of IL-1β stimulation, total RNA was extracted from F-NL cells using the NucleoSpin® RNA (Macherey Nagel, Düren, Germany) following the manufacturer's instruction. 500 ng of total mRNA was reverse transcribed to cDNA with the High-Capacity cDNA Reverse Transcription Kit (Applied Biosystems, Foster City, CA, USA). cDNA was then diluted 10 times and 5 μl were amplified using 1 μM primers and the KAPA SYBR® FAST qPCR Kit (Roche Diagnostics, West Sussex, UK). The relative quantitation was calculated with the 2^−ΔΔCT^ method using *GAPDH* as reference gene. The primer sequences used are: *GAPDH* forward 5′-ACAGTTGCCATGTAGACC-3′ and reverse 5′-TTTTTGGTTGAGCACAGG-3′; *COX*-*2* forward 5′-AAGCAGGCTAATACTGATAGG-3′ and reverse 5′-TGTTGAAAAGTAGTTCTGGG-3′; *ELAV1* forward 5′-GATCAGACTACAGGTTTGTC-3′ and reverse 5′-TTGAAACTGGTAATTGCCTC-3′; *TIA*-*1* forward 5′-GACTTTTTCACCATTTGGAC-3′ and reverse 5′-ACTTTCATGGGAATTGAACC-3′; *ZEP36* forward 5′-CAAGTAATCCCCT TTTCCAG-3′ and reverse 5′-CACCATCATGAATACTGAGC-3′.

### 2.3 Western blotting

After 24 h IL-1β stimulation, F-NL cells were lysed with RIPA buffer and frozen at −80 °C. Proteins were purified with high speed centrifugation and their concentration was determined by bicinchoninic acid (BCA) assay (ThermoFisher, Waltham, MA, USA). Proteins were diluted with 4× Laemmli buffer and boiled for 10 min. 10–20 μg of total protein were separated with SDS-PAGE and transferred into PVDF membrane. After 1 h blocking with 5% Blotto non-fat dry milk (Santa Cruz, Active Santa Cruz, CA, USA) in TBS 0.05% tween 20, the membranes were incubated with specific antibodies recognizing human forms of COX-2 (160112, Cayman Chemical), GAPDH (sc-47724, Santa Cruz), α-Tubulin (sc-8035, Santa Cruz), α-SMA (ab5694, Abcam, Cambridge, UK), COL1 (ab34710, Abcam) and TIA-1 (sc-1751, Santa Cruz). The optical densitometry (OD) of the protein bands was analyzed using Image Lab™ Software (Bio-Rad, Hercules, CA, USA). Data were normalized with the loading control GAPDH or α-Tubulin and fold changes from control condition were calculated.

### 2.4 Chromatin immunoprecipitation (ChIP)

F-NL cells were crosslinked using 1% formaldehyde after 72 h of treatment with SAHA in the presence or absence of TGF-β1 stimulation. ChIP assays were performed using the ChIP-IT Express Kit (53008, Active Motif, Carlsbad, CA, USA) as described previously [5]. Antibodies against acetylated histone H3 (06-599, Merck Millipore, Billerica, MA, USA), H3K27me3 (07-499, Merck Millipore), or normal rabbit IgG (12-370, Merck Millipore) were used for immunoprecipitation. 5 μl of purified immunoprecipitated-DNA was amplified using real-time PCR amplification with primers (0.5 μM) designed specifically for the *COX*-*2* promoter region (set A and B, Fig. 2A) and the KAPA SYBR® FAST qPCR Kit (KK4602, Roche Diagnostics). The primer sequences were the following: Set A forward 5′-ACAGCCTATTAAGCGTCGTCA-3′ and reverse 5′-CCGTGTCTGGTCTGTACGTC-3′, Set B forward 5′-AGCTTCCTGGGTTTCCGATT-3′ and reverse 5′-AGCCCATGTGACGAAATGACT-3′. ChIP data were analyzed using the Percent Input (% Input) method. Briefly, input Ct values corresponding to 1% of initial chromatin were adjusted to 100% and used to normalize Ct values of immunoprecipitated-DNA samples. ChIP PCR was performed in duplicate and the results were presented as mean ± SEM of three independent biological replicates (cell lines).

### 2.5 Bisulfite sequencing

Genomic DNA from F-NL cells was extracted with a standard procedure using phenol-chloroform. Bisulfite conversion of DNA (1 μg) was conducted with the EZ DNA Methylation™ Kit (D5001, Zymo Research, Irvine, CA, USA) following the manufacturer's instructions. *COX*-*2* promoter region was amplified using 1 μM primers (primer sequences: forward 5′-GGTAGGAAATTTTATATTGGTGATT-3′ and reverse 5′-CTCACCTATATAACTAAACRCCA-3′), 2.5 mM MgCl_2_, 1 μl HotStarTaq Plus DNA Polymerase (203601, Qiagen, Venlo, NL). Agarose gel electrophoresis was used to separate PCR products. DNA bands corresponding to *COX*-*2* promoter were dissected under UV-light and the DNA was extracted using the QIAquick Gel Extraction Kit (28704, Qiagen) following the manufacturer's instructions. DNA was then cloned using the pGEM®-T Easy Vector System II (A1380, Promega, Madison, WI, USA). Plasmids from 8 clones of each line were extracted using the QIAprep Spin Miniprep Kit (27104, Qiagen) following the manufacturer's instructions and were sequenced with a 3130xl ABI PRISM Genetic Analyzer (Life Technologies, Carlsbad, CA, USA).

### 2.6 COX-2 3′-UTR luciferase assay

*COX*-*2* 3′-UTR (2.75 Kb) was amplified using *COX*-*2* BAC DNA (Clone RP5 973-M2, Human BAC Resources) as a template. The forward and reverse PCR primers were designed to harbour *Sac*I and *Xho*I restriction sites, respectively. The primer sequences were the following: forward 5′-CTCTGAGCTCCAATGCAAGTTCTTCCCGCT-3′ and reverse 5′-CTCTCTCGAGTTTCCAACACAGTGTCGCAG-3′. 1 μg of purified PCR products was then digested using *Sac*I (R6061, Promega) and *Xho*I (R6161, Promega) restriction enzymes following the manufacturer's instructions. Digestion was then checked by agarose electrophoresis. Digested DNA was then ligated into the pmirGLO Dual-Luciferase miRNA Target Expression Vector (E1330, Promega) and cloned in JM109 competent cells (L200A, Promega). Plasmid harbouring *COX*-*2* 3′-UTR cloned downstream the firefly luciferase gene sequence was then amplified and purified.

F-NL cells were seeded into a 96 multiwell plate and pretreated with 5 μM SAHA or vehicle for 1 h prior to treatment with 2 ng/ml TGF-β1 for 48 h in complete DMEM. Cells were then transfected using FuGENE® HD Transfection Reagent (E2311, Promega) with the empty vector or the vector harbouring COX-2 3′-UTR. Briefly 250 ng of plasmid was incubated with 0.75 μl of transfection reagent for 15 min at room temperature in 10 μl final volume of serum-free DMEM. The transfection mix was then added to the well containing 90 μl of fresh serum-free and antibiotic-free DMEM and the cells were incubated at 37 °C, 5% CO_2_ in a humidified incubator for 3 h. The cells were then treated with SAHA and TGF-β1 for additional 48 h in serum-free DMEM and with 1 ng/ml IL-1β for the last 24 h. Firefly and *Renilla* luciferase activities were then measured using the Dual-Glo® Luciferase Assay System (E2920, Promega) following the manufacturer's instruction. The relative luciferase activity was calculated as the ratio between Firefly luciferase activity and *Renilla* luciferase activity. Reduced relative luciferase activity indicates decreased transcript stability and/or translation. Data were presented as mean ± SEM of three biological replicates.

### 2.7 mRNA stability assay

F-NL cells were pre-treated with 5 μM SAHA for 1 h prior to treatment with 2 ng/ml TGF-β1 for 72 h (48 h in complete DMEM followed by 24 h in serum-free DMEM). *COX*-*2* mRNA expression was induced by 1 ng/ml IL-1β for 2 h before the addition of 5 μM Actinomycin D (ActD) to stop mRNA synthesis. Total mRNA was isolated at 0, 2, 8, and 24 h post ActD treatment. *COX*-*2* mRNA expression was analyzed by qRT-PCR as described above. % change over control at 0 h ActD was then calculated.

### 2.8 siRNA transfection

F-NL cells were seeded in a 12 well plate (35,000 cells/well) in 250 μl of serum-free and antibiotic-free DMEM and transfected using HiPerFect Transfection Reagent (301704, Qiagen) and TIA-1 siRNA (SMARTpool: siGENOME TIA-1 siRNA, M-013042-02-0005, Darmacon Lafayette, CO, USA) or negative control siRNA negative control (1022076, Qiagen). Briefly, 60 nM *TIA-1* siRNA or control siRNA were incubated with 6 μl of transfection reagent for 10 min at room temperature in 250 μl final volume of media. The transfection mix was then added to the well and the cells were incubated at 37 °C, 5% CO_2_ in a humidified incubator for 3 h. After the addition of 500 μl complete DMEM to each well, the cells were then treated with TGF-β1 for 48 h. The transfection and TGF-β1 treatment were then repeated in serum-free and antibiotic-free DMEM and cells were treated with 1 ng/ml IL-1β for the last 24 h. Cells were then harvested for TIA-1 and COX-2 protein analysis by Western blotting.

### 2.9 PGE_2_ assay

PGE_2_ concentration in the culture medium was quantified using the Prostaglandin E_2_ ELISA Kit (514010, Cayman Chemical) following the manufacturer's instruction. The values obtained were adjusted with the total amount of protein per well.

### 2.10 Statistics

Data are presented as mean ± SEM from experiments using at least three different cell lines. Statistical analysis was performed using GraphPad Prism 6®. Student's *t*-test was performed to determine the significance of differences between two means. One way ANOVA was used to compare multiple conditions. A p value < 0.05 was accepted as statistically significant.

## 3 Results

### 3.1 SAHA prevents TGF-β1-induced downregulation of COX-2 in F-NL

**Fig. 1:**
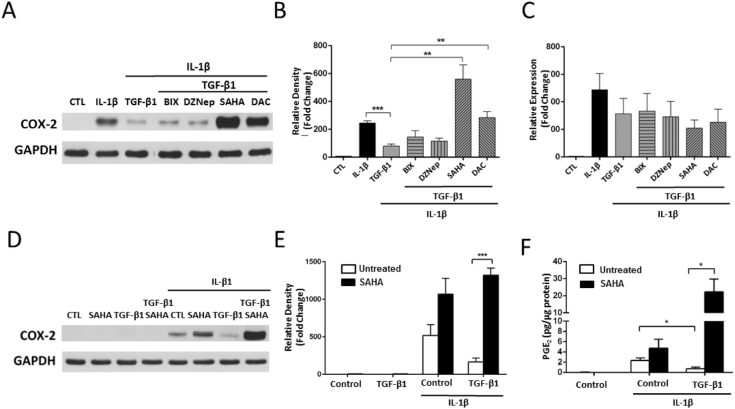
Effect of epigenetic inhibitors on COX-2 gene expression and PGE_2_ production. F-NL from 3 donors were pre-treated with the epigenetic inhibitors (100 nM BIX01294, 10 nM DZNep, 5 μM SAHA and 1 μM DAC) for 1 h prior to incubation with TGF-β1 (2 ng/ml) for 96 h, before further incubation with IL-1β (1 ng/ml) to induce COX-2 for 4 (for mRNA expression) and 24 (for protein expression) h. A, A representative Western blotting showing the effects of the epigenetic inhibitors on the protein expression of COX-2. B, Optical densitometry analysis of Western blotting bands for A. Data were normalized with the loading control GAPDH. Fold changes from control cells (CTL) were calculated. C, *COX*-*2* mRNA analysis by qRT-PCR using *GAPDH* as reference gene (2^−ΔΔCT^ method). Fold changes from control cells were calculated. D, A representative Western blotting showing the effects of SAHA on the protein expression of COX-2. E, Optical densitometry analysis of Western blotting bands for D. Data were normalized with the loading control GAPDH. Fold changes from control cells were calculated. F, PGE_2_ concentration in the media was analyzed by ELISA. All data are reported as mean ± SEM of three biological replicates (cell lines). *p < 0.05; **p < 0.01; ***p < 0.001.

To find out whether COX-2 could be downregulated in TGF-β1-induced lung fibroblast activation, F-NL cells were stimulated with TGF-β1 for 72 h. As reported before, TGF-β1-treated cells displayed a myofibroblast phenotype characterized by the upregulation of the fibrotic markers collagen 1 (COL1) and α-smooth muscle actin (α-SMA) compared with unstimulated control cells (Supplementary Fig. 1). COX-2 protein was not constitutively expressed in F-NL, but was induced by treatment with IL-1β for 24 h. However, in TGF-β1-treated cells, IL-1β-induced COX-2 expression was significantly reduced compared with cells treated with IL-1β alone (Fig. 1A and B). Among the tested epigenetic inhibitors, the pan-HDAC inhibitor SAHA and the DNA hypomethylating agent DAC prevented TGF-β1-induced COX-2 downregulation, but the G9a inhibitor BIX01294 (BIX) and the EZH2 inhibitor DZNep displayed no effect on COX-2 expression (Fig. 1A and B). *COX*-*2* mRNA was undetectable in control cells, IL-1β also induced a significant increase of *COX*-*2* mRNA in F-NL. A modest, but insignificant reduction of *COX*-*2* mRNA was observed following TGF-β1 treatment, but none of the epigenetic inhibitors caused any significant change of *COX*-*2* mRNA compared with TGF-β1 treatment alone (Fig. 1C). SAHA alone had no effect on COX-2 protein expression, but was also able to upregulate IL-1β-induced COX-2 protein expression in F-NL with or without TGF-β1 treatment (Fig. 1D and E). Consistent with COX-2 protein expression, IL-1β also increased the production of PGE_2_, a major antifibrotic product of COX-2 activity. However, the increase was significantly reduced by TGF-β1 treatment and SAHA was also able to enhance IL-1β-induced PGE_2_ production in F-NL treated with or without TGF-β1 (Fig. 1F). The results suggest that SAHA can prevent TGF-β1-induced downregulation of COX-2 protein expression and reduction of COX-2 activity (PGE_2_ production) during the process of lung fibroblast activation.

### 3.2 TGF-β1-induced COX-2 downregulation is not clearly associated with epigenetic modifications

**Fig. 2:**
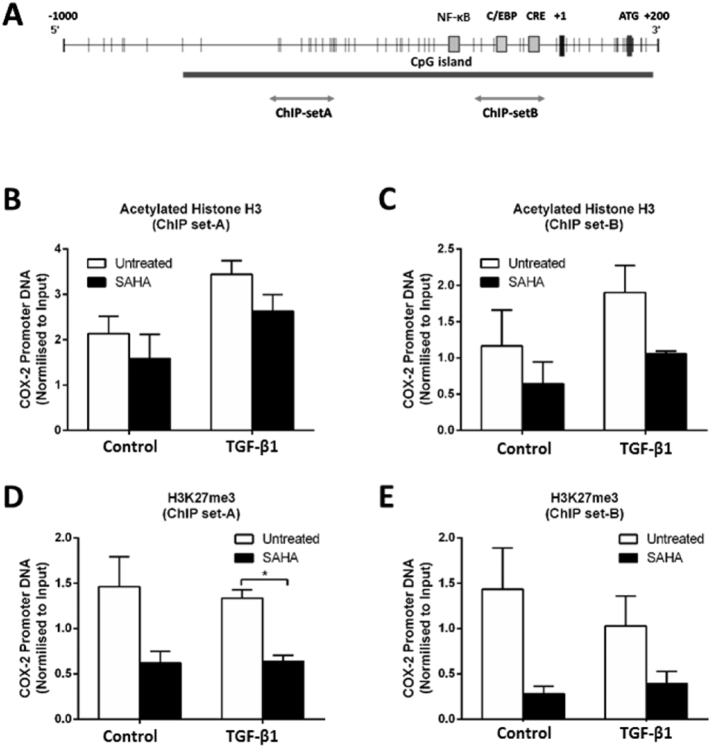
Effect of SAHA on histone modifications associated with the *COX*-*2* promoter. A, Schematic representation of *COX*-*2* promoter region identifying single CpG sites (vertical bar), binding sites for transcription factors NF-κB (Nuclear factor-κB), C/EBP (CCAAT/enhancer binding protein) and CRE (cAMP-response element), transcription start site (+1), translational coding site ATG and regions amplified by ChIP primers (ChIP-set A and ChIP-set B). B–E, F-NL from 3 donors were pre-treated with SAHA (5 μM) for 1 h prior to incubation with TGF-β1 (2 ng/ml) for 72 h. ChIP assay was performed using antibodies against acetylated histone H3 (B, C) and H3K27me3 (D, E) and the associated *COX*-*2* promoter DNA was detected by real-time PCR using ChIP-set A (B, D) and ChIP-set B (C, E) primers. Data are normalized to the input control and reported as mean ± SEM of three biological replicates. *p < 0.05.

Since previous studies have demonstrated association of histone modifications (e.g. histone deacetylation and H3K27me3) and DNA methylation with COX-2 repression in IPF and human gastric carcinoma [3,5,24], we went on to investigate the potential role of epigenetic modifications in TGF-β1-induced COX-2 downregulation in F-NL and whether these modifications could be altered by SAHA. Chromatin Immunoprecipitation (ChIP) analysis was performed using antibodies recognizing the acetylated form of histone H3 and H3K27me3 and two sets of primers (set A and B) amplifying the *COX*-*2* promoter regions highlighted in Fig. 2A. As pilot data showed no effect of IL-1β on the association of acetylated histone H3 and H3K27me3 with the *COX*-*2* promoter in TGF-β1-stimulated cells (data not shown), we decided to focus our attention on TGF-β1 and SAHA to assess their effect on histone modifications without any additional influence of IL-1β. Comparable results were obtained using the two ChIP primer sets (Fig. 2C–E). As shown in Fig. 2B and C, TGF-β1 stimulation induced a slight enrichment of acetylated H3 at the two regions of the *COX*-*2* promoter; pre-treatment with SAHA reduced H3 acetylation alone and prevented TGF-β1-induced increase, but the effect was not statistically significant. TGF-β1 stimulation did not have any effect on H3K27me3 association with the *COX*-*2* promoter, but pre-treatment with SAHA, either alone or in combination with TGF-β1, reduced H3K27me3 association, with significant reduction (p < 0.05) observed only with primer set A in TGF-β1-stimulated cells (Fig. 2D and E).

Bisulfite sequencing was then performed to identify the DNA methylation status of the *COX*-*2* promoter region (−692 to +168) in F-NL and to explore whether TGF-β1 could alter the DNA methylation. As shown in Supplementary Fig. 2, the CpG sites in the region of the *COX*-*2* promoter were mostly unmethylated in F-NL and TGF-β1 treatment did not markedly alter the methylation status of these CpG sites.

These observations suggest that the epigenetic modifications, including histone deacetylation, H3K27me3 and DNA methylation, are unlikely involved in the downregulation of COX-2 expression in TGF-β1-induced fibroblast activation under our experimental conditions and that the effect of SAHA on preventing COX-2 downregulation is not mediated by regulating epigenetic modifications at the *COX*-*2* promoter.

### 3.3 3′-UTR-mediated post-transcriptional control is involved in COX-2 regulation by SAHA

**Fig. 3:**
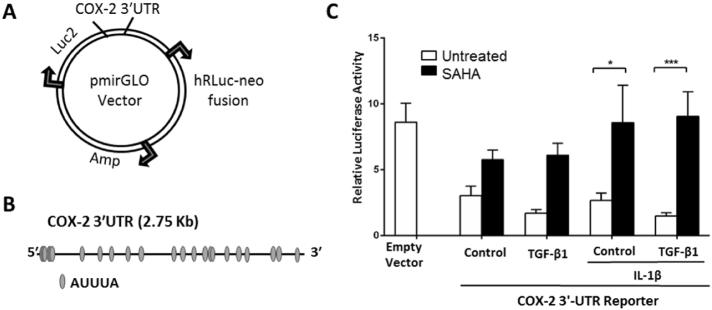
Effect of SAHA on *COX*-*2* 3′-UTR luciferase activity. A, Schematic representation of the luciferase vector used to study the effect of *COX*-*2* 3′-UTR on gene expression. *COX*-*2* 3′-UTR was cloned downstream the firefly luciferase gene (Luc2) in the pmirGLO Dual-Luciferase miRNA Target Expression Vector that harbours also the *Renilla* luciferase (hRLuc-neo fusion) as a control reporter to normalize data. B, Cloned *COX*-*2* 3′-UTR (2.75 Kb) with AUUUA sites, targets of ARE binding proteins. C, F-NL from 3 donors were pre-treated with SAHA (5 μM) for 1 h prior to incubation with TGF-β1 (2 ng/ml) for 48 h. The cells were then transfected with the empty vector or the vector harbouring *COX*-*2* 3′-UTR. SAHA and TGF-β1 treatments were repeated for additional 48 h, in combination with IL-1β (1 ng/ml) to induce *COX-2* for the last 24 h. Relative luciferase activity was calculated as the ratio between firefly luciferase activity and *Renilla* luciferase activity. Data are reported as mean ± SEM of three biological replicates. *p < 0.05; ***p < 0.001.

*COX*-*2* gene expression analysis highlights a lack of association between *COX*-*2* mRNA and protein levels, as COX-2 protein expression was increased by 7.9-fold when SAHA was administered in TGF-β1-treated F-NL (Fig. 1E), whereas the mRNA levels were comparable (Fig. 1C). ChIP analysis also suggests a lack of epigenetic modifying mechanisms in mediating the effect of SAHA on COX-2 expression (Fig. 2). To evaluate if post-transcriptional mechanisms of gene expression control that target the *COX*-*2* mRNA 3′-UTR could be responsible for the apparent discrepancy between COX-2 protein and mRNA expression by SAHA treatment, the *COX*-*2* mRNA 3′-UTR was cloned downstream a luciferase gene in the pmirGLO Dual-Luciferase miRNA Target Expression Vector (Fig. 3A). The vectors, empty or harbouring the 2.75 Kb *COX*-*2* 3′-UTR (Fig. 3B), were then transfected into F-NL cells treated with SAHA and TGF-β1.

The luciferase activity was therefore regulated post-transcriptionally by the *COX*-*2* 3′-UTR. Compared with cells transfected with empty vector, relative luciferase activity was reduced 2.8-fold in control cells transfected with the *COX*-*2* 3′-UTR with or without IL-1β (Fig. 3C), suggesting that the *COX*-*2* 3′-UTR can post-transcriptionally downregulate firefly luciferase protein expression (activity) in untreated F-NL. TGF-β1 treatment with or without IL-1β stimulation induced a further modest, but insignificant, reduction of the relative luciferase activity compared with control cells. In contrast, SAHA treatment induced a marked increase of relative luciferase activity, particularly when IL-1β was applied, 3.2-fold and 6.1-fold increases with respect to control cells (p < 0.05) and TGF-β1-treated cells (p < 0.001), respectively (Fig. 3C). The luciferase assay data suggest that COX-2 protein expression is regulated post-transcriptionally and that SAHA can act on the *COX*-*2* gene post-transcriptional machinery to increase IL-1β-induced COX-2 protein expression, possibly as a consequence of increased COX-2 translation and/or mRNA stability.

### 3.4 SAHA stabilizes COX-2 mRNA

**Fig. 4:**
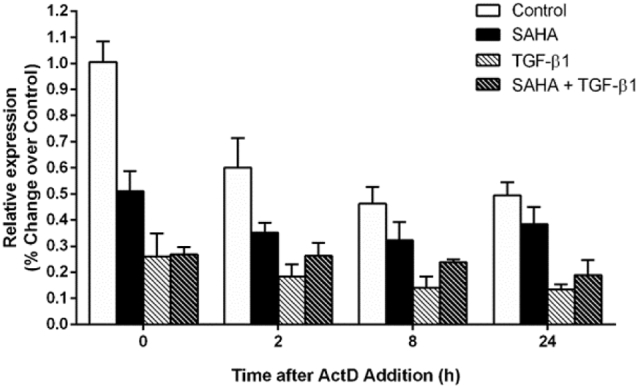
Effect of SAHA on *COX*-*2* mRNA stability. F-NL from 3 donors were pre-treated with SAHA (5 μM) for 1 h prior to incubation with TGF-β1 (2 ng/ml) for 72 h. *COX*-*2* mRNA expression was induced by incubation with IL-1β (1 ng/ml) to induce *COX-2* mRNA for 2 h prior the addition of ActinomycinD (ActD, 5 μM) to inhibit mRNA synthesis. *COX*-*2* mRNA level was evaluated by qRT-PCR using *GAPDH* as reference gene (2^−ΔΔCT^ method). Fold changes from control condition at 0 h ActD were calculated and data are reported as mean ± SEM of three biological replicates.

To identify if *COX*-*2* mRNA turnover was modified by SAHA, an actinomycin D (ActD) chase experiment was performed. F-NL cells were cultured following the usual schedule of treatment, but 5 μM ActD was added after 2 h stimulation with IL-1β. Total mRNA was extracted at 0, 4, 8, and 24 h after ActD addition. In control cells, IL-1β-induced *COX*-*2* mRNA was reduced to 59.7%, 46.0% and 49.2% at 2, 8, and 24 h respectively post ActD addition compared with 100% at 0 h post ActD addition, suggesting a natural degradation of IL-1β-induced *COX*-*2* mRNA in F-NL (Fig. 4). Compared with control cells, treatment with SAHA alone markedly reduced IL-1β-induced *COX*-*2* mRNA expression (0 h), and 68.8%, 63.0% and 75.2% *COX*-*2* mRNA was maintained at 2, 8, and 24 h post ActD addition compared with 100% at 0 h (SAHA alone), suggesting that SAHA may increase the stability of transcribed *COX*-*2* mRNA (Fig. 4). TGF-β1 treatment also reduced *COX*-*2* mRNA expression, but did not alter the natural degradation of *COX*-*2* mRNA as 70.7%, 54.1%, and 51.6% *COX*-*2* mRNA was maintained at 2, 8, and 24 h post ActD addition compared with 100% at 0 h (TGF-β1 alone), similar to control cells (Fig. 4). SAHA treatment did not alter TGF-β1-induced reduction on *COX*-*2* mRNA expression, but increased the stability of transcribed *COX*-*2* mRNA as 98.1%, 88.5% and 70.4% *COX*-*2* mRNA was maintained at 2, 8, and 24 h respectively post ActD addition compared with 100% at 0 h (SAHA + TGF-β1), but the effect of SAHA was not statistically significant (Fig. 4). These observations suggest that SAHA may cause a modest stabilization of *COX*-*2* mRNA, however, this effect is unlikely to provide a plausible explanation to the dissociation between SAHA-induced increase of COX-2 protein expression and unchanged mRNA expression in TGF-β1-treated F-NL and to the significant induction of *COX-2* 3′-UTR-regulated luciferase activity by SAHA over TGF-β1-treated F-NL.

### 3.5 SAHA induces the downregulation of the ARE binding protein TIA-1

**Fig. 5:**
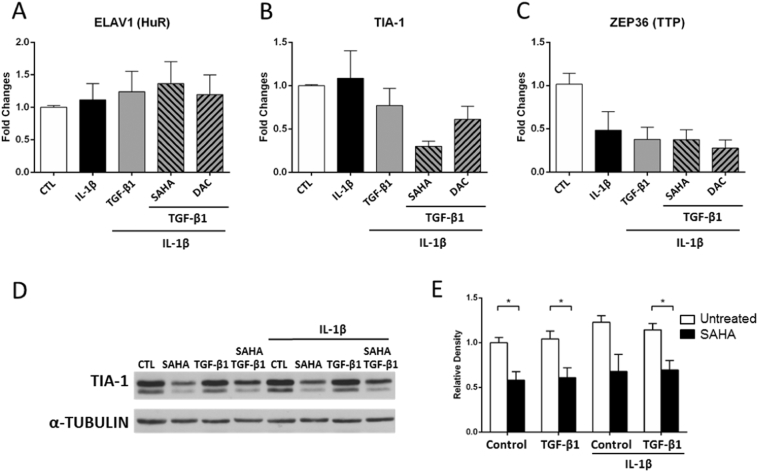
Effect of epigenetic inhibitors on ARE binding protein gene expression. F-NL from 3 donors were pre-treated with SAHA (5 μM) and DAC (1 μM) for 1 h prior to incubation with TGF-β1 (2 ng/ml) for 96 h before further incubation with IL-1β (1 ng/ml) to induce *COX-2* for the last 4 (for mRNA expression) and 24 (for protein expression) h. A–C, mRNA expression analysis of *HuR*, *TIA*-*1* and *TTP* was performed by qRT-PCR using *GAPDH* as reference gene (2^−ΔΔCT^ method). Fold changes from control condition (CTL) were calculated. D, A representative Western blotting showing the effect of SAHA on TIA-1 protein expression using α-Tubulin as loading control. E, Optical densitometry analysis of Western blotting bands. Data were normalized with the loading control α-tubulin. Fold changes from control condition were calculated. Data are reported as mean ± SEM of three biological replicates. *p < 0.05.

*COX*-*2* 3′-UTR harbours many AREs, targets of a family of proteins termed ARE-binding proteins. These proteins play an important role in the modulation of their target gene expression, by influencing mRNA turnover and translation. In order to evaluate the participation of ARE-binding proteins in *COX*-*2* regulation by SAHA in our cell system, we studied the effect of TGF-β1, SAHA and DAC on the mRNA expression of *ELAV1* (HuR), *TIA*-*1* and *ZEP36* (TTP) in F-NL cells in the presence of IL-1β. Compared with control cells, IL-1β and TGF-β1 stimulation had no effect on *ELAV1* (HuR) and *TIA*-*1* mRNA expression, pre-treatment with DAC did not alter *ELAV1* and *TIA*-*1* mRNA expression in TGF-β1-stimulated cells; however, pre-treatment with SAHA, but not DAC, markedly reduced *TIA*-*1*, but not *ELAV1* mRNA expression in TGF-β1-stimulated cells (Fig. 5A and B). IL-1β alone induced a downregulation of *ZEP36* and further treatment with TGF-β1, SAHA and DAC did not alter the downregulation (Fig. 5C). Consistent with mRNA expression, IL-1β and TGF-β1 stimulation had no effect on TIA-1 protein expression in F-NL, however, treatment with SAHA, either alone or together with TGF-β1 and IL-1β, significantly reduced TIA-1 protein expression in F-NL (Fig. 5D–E). Since TIA-1 has been reported to inhibit *COX*-*2* translation [23], its downregulation induced by SAHA is likely to be associated with the consistent upregulation of COX-2 protein expression (Fig. 1) and increased *COX*-*2* 3′-UTR reporter activity (Fig. 3).

### 3.6 TIA-1 repression via siRNA is associated with COX-2 upregulation

**Fig. 6:**
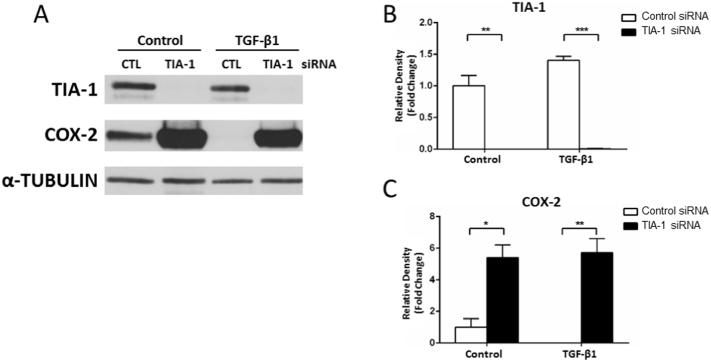
Effect of TIA-1 knockdown on COX-2 expression. F-NL from 3 donors were transfected with *TIA-1* siRNA or control siRNA (CTL) and then treated with or without TGF-β1 (2 ng/ml) for 48 h. The transfection and the treatments were then repeated for additional 48 h in combination with IL-1β (1 ng/ml) to induce COX-2 for the last 24 h. A, A representative Western blotting showing the effect of TIA-1 siRNA on TIA-1 and COX-2 protein expression. B–C, Optical densitometry analysis of Western blotting bands. Data were normalized with the loading control α-Tubulin. Fold changes from control condition treated with control siRNA were calculated and data are reported as mean ± SEM of three biological replicates. *p < 0.05; **p < 0.01, ***p < 0.001.

In order to explore if TIA-1 could play a role in the regulation of *COX*-*2* gene expression, a set of four siRNA probes specific for *TIA*-*1* were transfected in F-NL cells with or without TGF-β1 treatment, the cells were then treated with IL-1-β to induce COX-2, and the protein expression of TIA-1 and COX-2 was analyzed by Western blotting. The constitutive TIA-1 expression was not affected by TGF-β1 treatment, but was completely abolished by *TIA*-*1* siRNA in both control and TGF-β1-treated cells (Fig. 6A and B). The knockdown of *TIA*-*1* was accompanied by a significant upregulation of IL-1β-induced COX-2 protein expression in control cells and prevented TGF-β1-induced COX-2 downregulation (Fig. 6A and C). The effect of TIA-1 knockdown on COX-2 was similar to that of SAHA (Fig. 1D and E), suggesting that the post-transcriptional control induced by SAHA on COX-2 expression is largely mediated by the downregulation of TIA-1 and subsequent increase of *COX*-*2* mRNA translation.

## 4 Discussion

Repressed *COX*-*2* expression and deficient PGE_2_ have been shown to contribute to the activation of lung fibroblasts and the excessive deposition of collagen in pulmonary fibrosis. In fibroblasts obtained from fibrotic lungs, *COX*-*2* repression has been associated with epigenetic dysregulation characterized by reduced histone acetylation and increased H3K9 and H3K27 methylation at the *COX*-*2* promoter and epigenetic inhibitors targeting these histone modifications are able to restore *COX*-*2* gene expression and PGE_2_ production [3,5]. COX-2 downregulation and reduced PGE_2_ production are also associated with TGF-β1-induced lung fibroblast activation [4]. The main aim of this study was to investigate whether epigenetic inhibitors, could prevent COX-2 downregulation and reduced PGE_2_ production in TGF-β1-activated lung fibroblasts. Among the epigenetic inhibitors we tested, the pan-HDAC inhibitor SAHA was the most effective to prevent COX-2 downregulation induced by TGF-β1 in F-NL. To our surprise, the effect of SAHA on COX-2 was not mediated by regulating epigenetic modifications at the *COX*-*2* promoter, instead was mostly mediated by a novel TGF-β1-independent post-transcriptional mechanism, through the downregulation of the translational silencer of *COX*-*2* expression TIA-1.

We confirmed that TGF-β1-activated lung fibroblasts were associated with COX-2 downregulation and reduced PGE_2_ production as previously reported [4] and demonstrated that SAHA and the DNA demethylating agent Decitabine were able to increase COX-2 protein expression in response to IL-1β, but had no effect on the reduced *COX*-*2* mRNA expression. On the other hand, the G9a inhibitor BIX01294 and the EZH2 inhibitor DZNep had no effect on the expression of both COX-2 protein and mRNA, despite that global decreases of H3K9me3 and H3K27me3 were detected in cells treated with BIX01294 and DZNep, respectively (data not shown), suggesting that G9a and EZH2 are inhibited by the drugs, but the inhibition does not influence *COX*-*2* expression in TGF-β1-treated F-NL. This is different from fibroblasts derived from IPF patients [5] where G9a and EZH2 are the major actors driving COX-2 silencing. Further experiments focusing on SAHA revealed that SAHA alone had no effect on COX-2 protein expression, but increased IL-1β-induced COX-2 expression in cells not treated with TGF-β1, suggesting that SAHA can act on COX-2 expression independently of TGF-β1. Although the increase of IL-1β-induced COX-2 protein expression by SAHA was not significant compared with IL-1β alone and could be due to the variability among cell lines derived from different donors, there is a possibility that post-transcriptional regulation induced by SAHA (i.e. the downregulation of the *COX-2* translational silencer TIA-1) could lead to enhanced translation of IL-1β-induced *COX-2* mRNA. It is also interesting that, although both COX-2 expression and PGE_2_ production were reduced in TGF-β1-treated cells compared with control cells, the enhancing effect of SAHA on both, particularly PGE_2_ production, was stronger in TGF-β1-treated cells compared with control cells (Fig. 1F). Since PGE_2_ production downstream of COX-2 (immediate product PGH_2_) is influenced by the balance between the enzyme that promotes its synthesis (prostaglandin E synthase, PGES), the enzymes that reduce its synthesis by diverting PGH_2_ towards the synthesis of other PGs (e.g. synthases for PGI_2_, PGD_2_ and thromboxane A_2_ (TXA_2_)) and the enzyme that causes its degradation (15-hydroxyprostaglandin dehydrogenase (15-PGDH)), therefore, a strong linear correlation does not always exist between the level of COX-2 expression and the level of PGE_2_ production. In our current study, it is possible that SAHA may alter the balance of the enzymes influencing PGE_2_ production downstream of COX-2 and PGE_2_ degradation in TGF-β1-treated cells much more than control cells to favour exaggerated PGE_2_ production, but this needs to be explored by further studies.

Since *COX*-*2* silencing in F-IPF is critically associated with histone deacetylation and methylation [3,5], we then investigated whether changes in H3 acetylation and H3K27me3 state at the *COX*-*2* promoter contributed to COX-2 downregulation in TGF-β1-activated fibroblasts and whether the effect of SAHA was mediated by epigenetic regulation. Surprisingly, TGF-β1 stimulation induced a slight but insignificant increase of H3 acetylation at the *COX*-*2* promoter, which was slightly reduced by SAHA treatment, even though global H3 acetylation was increased (data not shown). The inhibition of histone acetylation at the *COX*-*2* promoter by SAHA is consistent with a previous study showing inhibition of histone H3 and H4 acetylation at the *osteopontin* gene promoter by another HDAC inhibitor trichostatin A (TSA) [25]. Furthermore, TGF-β1 stimulation did not cause any significant change of H3K27me3; however, the association of H3K27me3 with the *COX*-*2* promoter region covered by primer set-A was significantly inhibited by SAHA. As H3 acetylation and H3K27me3 are associated with gene expression and repression, respectively, the histone modifications we observed were inconsistent with reduced *COX*-*2* mRNA and protein expression, suggesting a dissociation between repressive histone modifications and COX-2 downregulation in TGF-β1-activated fibroblasts. This is further supported by the fact that the G9a and EZH2 inhibitors had no effect on the COX-2 downregulation, which also suggests that the effect of SAHA on COX-2 is unlikely mediated by reducing H3K27me3.

It is interesting that methylation of the CpG sites at the *COX*-*2* promoter was not different between TGF-β1-treated and control F-NL cells. This is consistent with a previous report [26] and suggests that DNA methylation is also not involved in the COX-2 downregulation in TGF-β1-activated fibroblasts, despite that it is involved in COX-2 repression in fibrotic lung fibroblasts [5]. However, increased COX-2 protein expression was observed with the demethylating agent DAC in TGF-β1-activated fibroblasts. Although it is possible that DAC may upregulate COX-2 expression indirectly by demethylating other hypermethylated and downregulated targets, such as the transcriptional regulator chromosome 8 open reading frame 4 (*c8orf4*) [26], this is unlikely in our current study as *COX*-*2* mRNA expression was not enhanced by DAC.

This dissociation between COX-2 downregulation and epigenetic repression in TGF-β1-activated fibroblasts is clearly different from the *COX*-*2* silence in fibrotic lung fibroblasts, which is associated with histone deacetylation and H3K9 and H3K27 methylation as well as DNA methylation [3,5]. The difference may be explained by the length of TGF-β1 treatment, since TGF-β1 could both stimulate and repress COX-2 expression depending on how long the treatment is maintained. We previously reported that TGF-β1 induced both COX-2 mRNA and protein expression for up to 24 h in F-NL and an increased association of H3 acetylation with the *COX*-*2* promoter for up to 4 h, similar to the effect of IL-1β [3]. We showed in this study that TGF-β1 treatment for 72 h did not induce COX-2 expression in the same cells on its own and significantly reduced IL-1β-induced COX-2 expression and that this downregulation was not clearly associated with epigenetic repression. These observations suggest a shift from an epigenetically active to epigenetically neutral state at the *COX*-*2* promoter from 24 h to 72 h of TGF-β1 treatment in F-NL. It is conceivable that a further shift to an epigenetically repressive state at the *COX*-*2* gene promoter may occur if TGF-β1 treatment is prolonged beyond 72 h. It is also possible that TGF-β1 treatment alone may not be sufficient to induce epigenetic silencing of the *COX*-*2* gene, although it is enough to induce COX-2 downregulation and fibroblast activation.

Since COX-2 downregulation is not primarily regulated by chromatin-dependent mechanisms in TGF-β1-activated fibroblasts and SAHA upregulates COX-2 protein expression, but not mRNA expression, it is possible that the effect of SAHA on COX-2 may be mediated by a post-transcriptional mechanism. Our data using a *COX*-*2* mRNA 3′-UTR luciferase reporter assay support this hypothesis, since an increased relative luciferase activity was detected in SAHA-treated cells, which indicates increased *COX*-*2* mRNA stabilization and/or translation. Further studies on *COX*-*2* mRNA stability shows that *COX*-*2* mRNA decay was slowed down by SAHA compared with TGF-β1-treated cells; however, the effect was not statistically significant. Furthermore, IL-1β-induced *COX*-*2* mRNA in SAHA-treated cells remained low, similar to that in cells treated with TGF-β1 alone, and SAHA showed no effect on the expression of *HuR* and *TTP*, ARE binding proteins promoting *COX*-*2* mRNA stabilization [27] and degradation [14], respectively, although *TTP* expression was reduced by IL-1β. These observations could not explain the discrepancy between high level of COX-2 protein and low level of mRNA, as well as the significantly increased *COX*-*2* mRNA 3′-UTR reporter activity in SAHA-treated TGF-β1-activated fibroblasts and strongly suggest that mRNA stability is unlikely to contribute to COX-2 upregulation by SAHA and that an increased mRNA translation could play a more significant role. Interestingly, TIA-1, an ARE-binding translational silencer of COX-2 expression [15,17], was downregulated at both mRNA and protein levels by SAHA, either alone or with TGF-β1, and independently of IL-1β stimulation, suggesting that TIA-1 is likely to play a role in SAHA-mediated COX-2 upregulation. Indeed, TIA-1 depletion via siRNA resulted in a significant increase of IL-1β-induced COX-2 protein expression in both control and TGF-β1-treated F-NL, which is in line with COX-2 upregulation associated with TIA-1 downregulation induced by SAHA. This finding is consistent with previous reports showing increased translation of IL-1β-induced *COX*-*2* mRNA by TIA-1 depletion in human osteoarthritis chondrocytes [28] and increased COX-2 protein expression, but not *COX*-*2* transcription or mRNA turnover in TIA-1 null macrophages [15] and fibroblasts [17]. Furthermore, binding of TIA-1 to the proximal region of the 3′-UTR of *COX*-*2* following IL-1β has also been demonstrated by electrophoretic mobility shift assay (EMSA) in renal mesangial cells [29] and defective RNA binding of TIA-1 has been shown to promote COX-2 expression in colon cancer cells [17]. These observations and our data corroborate the role of TIA-1 as an inhibitor for *COX*-*2* mRNA translation and strongly suggest that the effect of SAHA on COX-2 upregulation is largely mediated through the downregulation of TIA-1 and increased translation of *COX*-*2* mRNA. Notably, TIA-1 depletion in F-NL also resulted in a significant downregulation of the fibrotic markers α-smooth muscle actin (α-SMA) and collagen 1 (data not shown), suggesting TIA-1 could be critically involved in the regulation of other antifibrotic and profibrotic genes and could be a potential target for antifibrotic intervention. This is also consistent with the antifibrotic effect of SAHA [30]. It is possible that the antifibrotic effect of SAHA could be partially mediated through the downregulation of TIA-1, although how SAHA regulates the downregulation remains to be explored. It is also possible that non-coding RNAs, such as microRNAs (miRNAs), may play a role in mediating the effect of SAHA on COX-2 and TIA-1, since some miRNAs, such as miRNA-199a, miRNA-145a and miR-26a, have been reported to target and inhibit specifically *COX*-*2* [31,32] and HDAC inhibitors, such as SAHA, can modulate the expression of miRNAs implicated in fibrosis, such as miR-15a, miR-16 and miR-29b and Let-7b [[33], [34], [35]]. Moreover, *TIA*-*1* mRNA 3′-UTR has been indicated as a putative target of some of the previously reported miRNAs involved in fibrosis, such as miR-16, miR-15, and miR-26b, by the experimentally validated microRNA-target interactions database (miRTarBase, http://mirtarbase.mbc.nctu.edu.tw/index.php.). Therefore, TIA-1 downregulation associated with SAHA treatment could be mediated post-transcriptionally by miRNAs.

It is worth noting that the effect of SAHA on COX-2 upregulation in lung fibroblasts appears to be independent of the effect of TGF-β1 on COX-2 downregulation. This is evidenced by the observations that SAHA upregulated IL-1β-induced COX-2 protein expression and *COX-2* mRNA 3′-UTR-controlled reporter gene expression with or without the presence of TGF-β1 and that SAHA downregulated *TIA-1* mRNA and protein expression, but TGF-β1 had no effect. In addition, we also found that SAHA did not have any effect on the phosphorylation (activation) of SMAD2/3 (data not shown), the intracellular proteins that transduce extracellular signals from TGF-β1. Therefore, the effect of SAHA on COX-2 in lung fibroblasts appears not mediated by interfering with TGF-β1 signalling or directly antagonizing the effect TGF-β1 on COX-2 downregulation, although as a net effect SAHA did prevent COX-2 downregulation in TGF-β1-activated fibroblasts. The molecular mechanisms underlying the COX-2 downregulation by TGF-β1 are unclear so far.

In conclusion, our data demonstrate that SAHA can upregulate COX-2 expression and prevent COX-2 downregulation in TGF-β1-activated fibroblasts. The effect is TGF-β1-independent and chromatin-independent and is mostly mediated by a novel post-transcriptional mechanism through the downregulation of the translational silencer of COX-2 expression TIA-1. This novel mechanism may represent a promising strategy to restore the expression of COX-2 and other antifibrotic genes in pulmonary fibrosis.

## Funding

This work was supported by Medical Research Council (grant number MR/K003259/1).

## Author contributions

AP and LP designed the study. AP performed the experiments and analyzed the data. OB provided technical assistance, contributed to the design of the experiments and critically reviewed the paper. AP and LP wrote the paper. LP, GJ and AK provided the funding and critically reviewed the paper. All authors reviewed the results and approved the final version of the manuscript.

## Transparency document

Transparency document. Image 1

## Appendix A Supplementary data

Supplementary Fig. 1 Effect of TGF-β1 on F-NL phenotype change. F-NL from 2 donors (F-NL1 and F-NL2) were treated with 2 ng/ml TGF-β1 for 96 h. The fibrotic markers, collagen 1 (COL1) and α-smooth muscle actin (α-SMA) were detected by Western blotting in control (CTL) and TGF-β1-treated cells (TGF-β1). GAPDH was used as loading control. Supplementary Fig. 2. DNA methylation status of *COX*-*2* promoter in F-NL. Genomic DNA from F-NL and F-NL treated with TGF-β1 (2 ng/ml) for 96 h was analysed by Bisulfite sequencing. Each CpG site (vertical bar) is identified by squares corresponding to the different sequenced clones. A white or black square indicates an unmethylated or methylated CpG site in the specific clone, respectively. Image 2
