# Supplementary material for: Suberanilohydroxamic acid prevents TGF-β1-induced COX-2 repression in human lung fibroblasts post-transcriptionally by TIA-1 downregulation
Source: Biochim Biophys Acta. 2018 May;1861(5):463–72. doi: 10.1016/j.bbagrm.2018.03.007 (PMC5910054; doi:10.1016/j.bbagrm.2018.03.007)
Supplement: Supplementary file 2 — Supplementary Fig. 1 Effect of TGF-β1 on F-NL phenotype change. F-NL from 2 donors (F-NL1 and F-NL2) were treated with 2 ng/ml TGF-β1 for 96 h. The fibrotic markers, collagen 1 (COL1) and α-smooth muscle actin (α-SMA) were detected by Western blotting in control (CTL) and TGF-β1-treated cells (TGF-β1). GAPDH was used as loading control. Supplementary Fig. 2. DNA methylation status of COX-2 promoter in F-NL. Genomic DNA from F-NL and F-NL treated with TGF-β1 (2 ng/ml) for 96 h was analysed by Bisulfite sequencing. Each CpG site (vertical bar) is identified by squares corresponding to the different sequenced clones. A white or black square indicates an unmethylated or methylated CpG site in the specific clone, respectively. [file mmc2.docx]

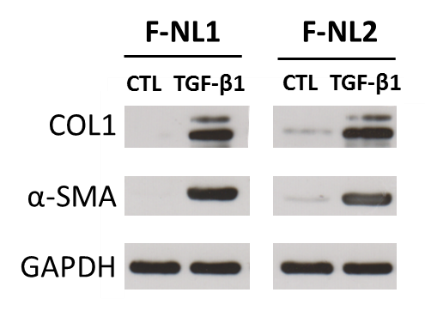


SUPPLEMENTARY FIGURE 1. **Effect of TGF-β1 on F-NL phenotype change.** F-NL from 2 donors (F-NL1 and F-NL2) were treated with 2 ng/mL TGF-β1 for 96 hrs. The fibrotic markers, collagen 1 (COL1) and α-smooth muscle actin (α-SMA) were detected by Western blotting in control (CTL) and TGF-β1-treated cells (TGF-β1). GAPDH was used as loading control.


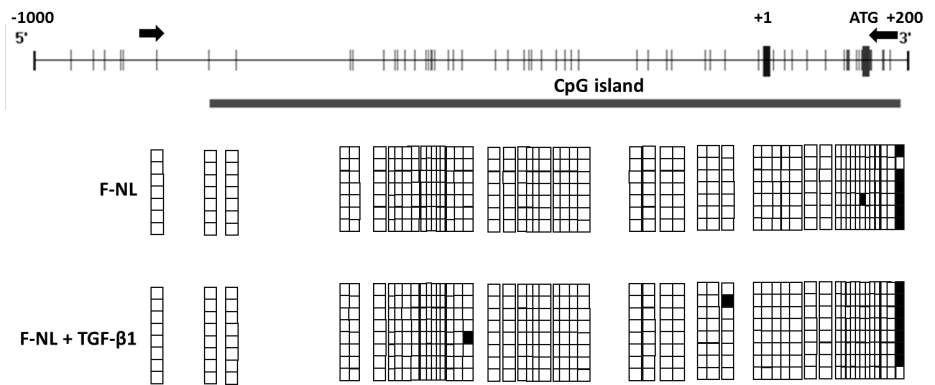


SUPPLEMENTARY FIGURE 2. **DNA methylation status of *COX-2* promoter in F-NL.** Genomic DNA from F-NL and F-NL treated with TGF-β1 (2 ng/ml) for 96 hrs was analysed by Bisulfite sequencing. Each CpG site (vertical bar) is identified by squares corresponding to the different sequenced clones. A white or black square indicates an unmethylated or methylated CpG site in the specific clone, respectively.
